# Why does the probe value effect emerge in working memory? Examining the biased attentional refreshing account

**DOI:** 10.3758/s13423-022-02056-6

**Published:** 2022-01-28

**Authors:** Amy L. Atkinson, Klaus Oberauer, Richard J Allen, Alessandra S. Souza

**Affiliations:** 1 Department of Psychology, Lancaster University, Bailrigg, Lancaster, LA1 4YW UK; 2 School of Psychology, University of Leeds, Leeds, LS2 9JT UK; 3 School of Languages, Cultures and Societies, University of Leeds, Leeds, LS2 9JT UK; 4 Department of Psychology, Cognitive Psychology Unit, University of Zurich, Binzmühlestrasse 14/22, 8050 Zurich, Switzerland; 5 Faculty of Psychology and Education Sciences, University of Porto, Rua Alfredo Allen, 4200-135 Porto, Portugal

**Keywords:** Prioritization, Attentional refreshing, Visual working memory

## Abstract

People are able to prioritize more valuable information in working memory. The current study examined whether this value effect is due to the items of greater value being refreshed more than lower-value items during maintenance. To assess this possibility, we combined a probe value manipulation with a guided-refreshing procedure. Arrays of colored shapes were presented, and after a brief delay, participants reported the color of one randomly probed shape on a continuous color wheel. To manipulate probe value, one item was indicated as more valuable than the rest prior to encoding (i.e., worth more notional points), or all items were indicated as equally valuable. To guide refreshing, in some trials, two arrows were presented during maintenance, each arrow cueing the spatial location of one item. Participants were told to “think of” (i.e., refresh) the cued item. If value boosts are driven by attentional refreshing, cueing an item to be refreshed should enhance performance for items that are of low or equal value, but not items of high value, as these items would be refreshed regardless of the cue. This pattern of outcomes was observed, providing support for the hypothesis that attentional refreshing at least partially accounts for probe value effects in working memory.

Working memory (WM) allows a limited amount of information to be temporarily stored in a state of heightened accessibility for use in ongoing processing (Cowan, 2017). As items often differ in their value or goal relevance (Oberauer & Hein, 2012; Souza & Oberauer, 2016), one must be able to prioritize certain representations to succeed in WM tasks. Indeed, research has revealed that individuals can direct their attention in WM based on visual cues (e.g., Loaiza & Souza, 2018; Rerko et al., 2014; Souza & Oberauer, 2016) and probe probability (where participants are informed at the start of the block that one particular item—for instance, identified by its serial position—is most likely to be tested; e.g., Atkinson et al., 2018; Gorgoraptis et al., 2011).

There is also evidence that individuals can prioritize more “valuable” information in WM. Value can be induced by monetary rewards (e.g., Klyszejko et al., 2014) or by simply offering notional points (see Hitch et al., 2020, for a review). In the latter paradigm, participants are presented with series of items to remember for a brief period. Before encoding, they are told that one item is worth a higher reward than the other items. Performance at the more valuable item is then compared to performance at the same serial position in a condition in which all items are of equal value (e.g., Atkinson et al., 2018; Atkinson et al., 2019) or a condition in which a different serial positions is more valuable (e.g., Hu et al., 2014; Hu et al., 2016). Individuals are better able to remember items worth a high reward than items worth a lower reward. This has been observed across various age groups (e.g., Allen et al., 2021; Atkinson et al., 2019), modes of presentation (e.g., Allen & Ueno, 2018; Hu et al., 2014), retrieval methods (Hu et al., 2014; Sandry et al., 2014), and study materials (e.g., Atkinson et al., 2021; Sandry et al., 2014).

What drives the probe value effect in WM? Hu et al. (2016) found that the value boost was drastically reduced or abolished when participants completed a cognitively demanding concurrent task during encoding and maintenance. This suggests that the value effect results from mechanisms during one (or both) of these stages. Two possibilities have been proposed. First, it has been suggested that the effect may emerge due to a biased attentional refreshing procedure, whereby the individual “thinks of” the more valuable item more during the retention interval, relative to the other items (Atkinson et al., 2018; Hitch et al., 2018; Sandry et al., 2014). The second possibility is that the probe value boost may result from differential encoding of high-value and low-value items (Sandry et al., 2014), with high-value items potentially encoded more strongly. In the present work, we examined the extent to which the value effect is due to preferential attentional refreshing.

Souza et al. (2015) developed a method to study attentional refreshing in WM. Participants were asked to briefly remember arrays of colored circles for a brief period and then to reproduce the color of one item by selecting it on a color wheel. During the retention interval, arrows cued the spatial location of some items. Participants were told to “think of” (i.e., refresh) the cued items. With this procedure, some circles were not cued to be refreshed during the retention interval, some were cued once, and other items were cued twice. Recall error decreased monotonically as the number of refreshes increased, suggesting that preferentially attending to some items during the retention interval improves WM performance.

Accordingly, the current study aimed to leverage the directed refreshing procedure developed by Souza et al. (2015) to investigate whether probe value effects rely on attentional refreshing. The study was conducted as an international collaboration between the University of Leeds (UK) and the University of Zurich (Switzerland). A secondary aim of the study was therefore to replicate the basic probe value and directed refreshing manipulations across different laboratories.

## The present study

Arrays of four colored shapes were presented, with one item probed following a brief delay. Participants had to select the color of this item on a continuous color wheel. Before item presentation, participants were either told that one of the items was relatively more valuable than the rest (i.e., worth 4 points, whereas the other items were worth 1 point), or that all items were equally valuable (i.e., all worth 1 point). This formed three probe value conditions: high value (i.e., the item probed was worth 4 points), equal value (i.e., all items were worth 1 point), and low value (i.e., one item was worth 4 points, but one of the low-value items was tested). In some trials, a sequence of two arrows was presented during the maintenance phase, with each arrow cueing the location of a different item. Participants were asked to “think of” the item the arrow pointed towards for the entire time the arrow was on-screen. In other trials, no arrows were presented. This created three directed refreshing conditions: cued (the tested item had been cued during maintenance), uncued (the tested item had not been cued), and none cued (no arrows were presented).

Of particular interest was whether an interaction would emerge between probe value and directed refreshing. If the probe value effect and the refreshing benefit arise from different mechanisms, these manipulations should be additive, leading to a refreshing benefit for high-value items as well as for equal-value and low-value items. In contrast, if probe value effects reflect biased attentional refreshing, the cueing boost for the high-value item should be reduced or absent (as this item would already be prioritized for refreshing). This would result in an interaction between probe value and directed refreshing, whereby equal-value and low-value items should receive a performance boost when they are cued to be refreshed, whereas high-value items would experience a smaller boost or no boost. However, cueing another item would draw refreshing away from the high-value item, incurring a cost for the high-value uncued item.

Another novel contribution of the present study was to examine how probe value manipulations change parameters reflecting the quantity and quality of the representations in WM. Data from the continuous color reproduction task can be modelled using mixture models (Bays et al., 2009; Zhang & Luck, 2008) that yield parameters reflecting the probability of recalling the tested item or of recalling a nontested item (as opposed to guessing). In addition, the model assumes that the memory items can be retrieved with different levels of precision (reflecting the fidelity of the representation in WM). Souza et al. (2015) reported that directed refreshing increases the accessibility of the refreshed item in WM, but not its precision. Such analysis has not yet been performed to investigate the theoretical parameters underlying probe value effects. As attentional refreshing is considered to enhance accessibility of items in WM (Camos et al., 2018; Souza et al., 2015; Vergauwe & Langerock, 2017), one would expect high-value items to have a greater probability of being retrieved relative to equal-value and low-value items if a biased refreshing process drives such effects.

## Method

### Participants

Forty participants completed the study in total (*M*_age_ = 23.20 years, *SD* = 3.78 years, 23 females, 11 males, six unknown), with 20 participants tested at the University of Leeds, UK (*M*_age_ = 22.45 years, *SD* = 3.46, 15 females, four males, one unknown) and 20 participants tested at the University of Zurich, Switzerland (*M*_age_ = 23.95 years, *SD* = 4.03, eight females, seven males, five unknown). Participants had normal or corrected-to-normal vision and no color-blindness. Participants were either native English speakers (University of Leeds) or native German speakers (University of Zurich). Participants were reimbursed for their time with cash (£20 in the UK and 45 CHF in Switzerland). The amount of money offered was considered similar based on the differences in the cost of living in the two countries and was unrelated to task performance. Ethical approved was granted by the School of Psychology Ethics Committee at the University of Leeds. The study was also conducted in accordance with the regulations of the Ethics Committee of the Faculty of Arts and Social Sciences at the University of Zurich.

### Design, materials, and procedure

The study employed a 3 (probe value: high, equal, low) × 3 (directed refreshing: cued, uncued, none cued) repeated-measures design. The probe value and directed refreshing trials were intermixed. In the main analysis, testing site was also entered as a between-subjects variable with two levels (Leeds, Zurich), to assess whether the effects were consistent across laboratories.

The task was completed as two sessions on different days, each lasting approximately 75–90 minutes. Participants completed 300 experimental trials during each session (600 trials in total). There were 120 equal-value trials and 480 trials where one item differed in value relative to the other items. Given that high-value and low-value items were equally likely to be tested, there were 120 trials in which a high-value item was tested, and 360 trials in which one of the low-value items was tested. In the equal-value and high-value conditions, each directed refreshing condition was tested 40 times. In the low-value condition, each directed refreshing condition was assessed 120 times. Within each of these cells, the four spatial locations were equally likely to be tested.

As the task was relatively complicated, participants completed practice trials for each element of the task separately in the first session. Participants first completed 10 practice trials in which no items were cued, but the items differed in value. They then completed 10 practice trials in which all items were equally valuable, but directed refreshing was manipulated. Finally, participants completed 15 practice trials in which both probe value and directed refreshing were manipulated. In the second session, participants completed the final practice block only, whereby probe value and directed refreshing were both manipulated.

**Fig. 1:**
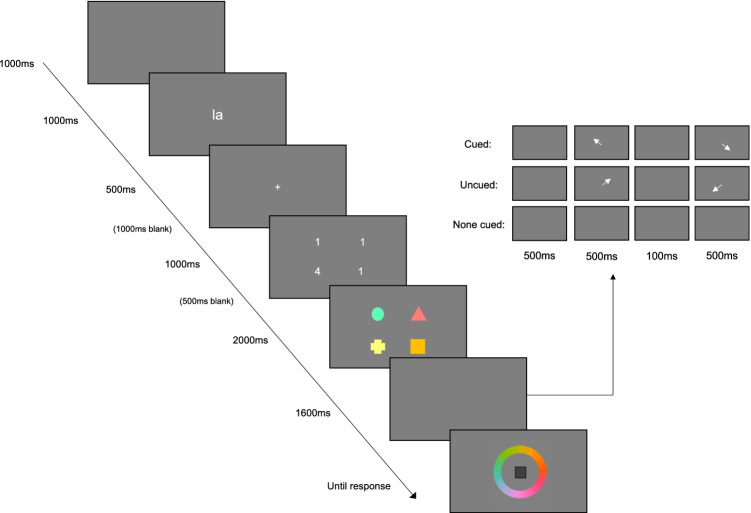
The experimental paradigm used. The array with numbers illustrates the probe value manipulation. The inset illustrates the directed refreshing procedure. In cued trials, the tested item was one of the cued items. In uncued trials, the tested item was one of the not-cued items. In none-cue trials, the screen remained blank for the whole retention interval. Displays are not draw to scale

The experimental paradigm used is displayed in Fig. 1. Each trial began with a blank screen presented for 1,000 ms, followed by the word “la” for a further 1,000 ms. Participants were asked to repeat this until the retrieval phase to disrupt verbal recoding (Baddeley, 1986). To ensure compliance, participants were either monitored during study administration (Leeds) or voice recordings were taken and checked retrospectively (Zurich).^1^ Next, a fixation cross was presented for 500 ms, followed by a blank screen for 1,000 ms. Point values were then presented on-screen for 1,000 ms, followed by a blank screen for 500 ms. The array of four to-be-remembered colored shapes was then presented for 2,000 ms on a grey background. In each array, a circle, square, triangle, and cross were presented (each measuring ~1.5°) at one of four spatial locations positioned at the corners of a 3.5° square located at the center of the screen. The colors of each shape were randomly selected from 360 values, evenly spaced around a circle in the CIELAB color space (L = 70, a = 20, b = 38, radius = 60).

Offset of the memory array was followed by a retention interval of 1,600 ms. In the cued and uncued conditions, the retention interval started with a blank screen (500 ms). This was followed by two arrows (ca. 1°), each presented for 500 ms and separated by a blank screen of 100 ms. In the none-cued condition, the screen remained blank for the entire 1,600 ms. One of the shapes was then presented in the center of the screen in dark grey, surrounded by a color wheel. The color wheel was presented as an annulus with inner radius of 25% of the screen height and an outer radius of 33% of the screen height, with a random rotation on every trial. Participants adjusted the color of the shape until it matched the color of the item during encoding. They responded by clicking on a color on the wheel.

During the instructions, participants were informed that the numbers appearing before the items denoted the point value of the item displayed at that spatial location (e.g., 4 = 4 points). Participants were told they would collect that number of points if they responded accurately, but the level of accuracy required was not specified. This was done to ensure that participants responded as accurately as possible. Participants were told to try to collect as many points as possible, although these were notional and unrelated to any reward (e.g., monetary reward). In line with previous studies, participants were not given feedback about the number of points collected (e.g., Atkinson et al., 2018; Hitch et al., 2018).

Participants were also told to pay attention to the arrows presented during the retention interval and to think of the item that appeared at the spatial location the arrow pointed towards for the entire time the arrow was on-screen. They were informed that neither the point values nor the arrows predicted which item would be tested. The instructions were presented in English at the University of Leeds and German at the University of Zurich.

Data, task scripts, and other materials are available at https://osf.io/gwtb9/. 

### Data analysis

The deviation between the correct color and the color selected was calculated, which ranged between −180° to 180°. The main dependent variable of interest was the absolute value of the deviation, referred to as recall error hereafter. The data were analyzed using both frequentist and Bayes factor (BF) analysis. BF analysis computes the strength of evidence for the presence (or absence) of an effect and can therefore be used to assess equivalence between conditions or groups. The Bayesian analyses of variance (ANOVAs) were run using the default priors (Rouder et al., 2012) of the BayesFactor package (Morey et al., 2018) implemented in R (R Core Team, 2018), with the number of iterations of the Markov chain Monte Carlo (MCMC) set at 500,000. The model with the highest likelihood is reported, as well as BFs for individual main effects and interactions. When appropriate, follow-up BF *t* tests were then conducted. A BF_10_ value above 1 provides evidence of an effect, whereas a BF_10_ below 1 (and a BF_01_ = 1/BF_10_, larger than 1) provides evidence of no effect. For the frequentist analysis, post hoc comparisons were corrected using Bonferroni–Holm.

The data were also fit using a Bayesian implementation (Oberauer et al., 2017) of the three-component mixture model (Bays et al., 2009) to establish whether the probe value and directed refreshing effects reflect an increased probability of recalling the target item, increased precision, or a decreased probability of recalling a nontarget item. The model was fit using four chains, each with 25,000 samples. Five thousand samples from each chain were discarded as warm-ups, leaving a total of 80,000 samples. MCMC chain convergence was assessed using the Gelman–Rubin $\hat{\mathrm{R}}$ statistic (Gelman & Rubin, 1992). All $\hat{\mathrm{R}}$ values were between 1.00 and 1.01, reflecting good convergence.

## Results

**Fig. 2:**
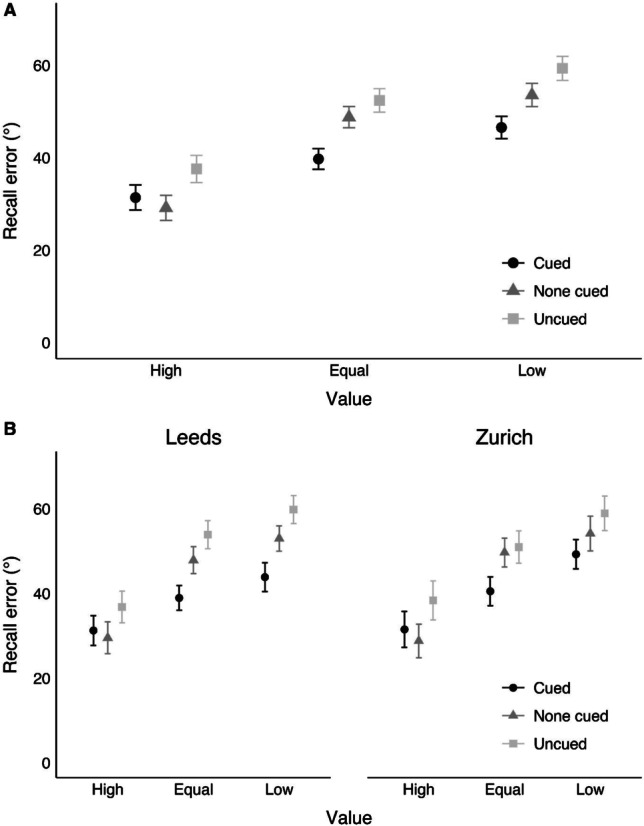
Mean recall error as a function of probe value and directed refreshing (**a**), and probe value, directed refreshing, and test site (**b**). Lower values reflect better performance. Error bars denote standard error

Mean recall error as a function of value and directed refreshing is displayed in Fig. 2a, whereas mean recall error as a function of probe value, directed refreshing, and test site is displayed in Fig. 2b. The 3 (probe value) × 3 (directed refreshing) × 2 (test site) mixed ANOVA revealed a significant main effect of probe value, *F*(1.18, 44.99) = 65.92, *p* < .001, η_p_^2^ = .63; BF_10_ > 10,000. Post hoc comparisons revealed significant differences between the items associated with a high value and low value (*p* < .001; BF_10_ > 10,000), high value and equal value (*p* < .001; BF_10_ > 10,000), and equal value and low value (*p* < .001; BF_10_ > 10,000). A significant main effect of cueing also emerged, *F*(1.33, 50.60) = 31.30, *p* < .001, η_p_^2^ = .45; BF_10_ > 10,000, with significant differences between the cued and uncued conditions (*p* < .001; BF_10_ > 10,000), the cued and none-cued conditions (*p* < .001; BF_10_ = 1,392.20), and the none-cued and uncued conditions (*p* < .001; BF_10_ > 10,000). A significant interaction between probe value and directed refreshing was observed, *F*(3.09, 117.26) = 7.79, *p* < .001, η_p_^2^ = .17; BF_10_ = 5.15. There was no significant main effect of test site, *F*(1, 38) = 0.04, *p* = .852, η_p_^2^ < .01; BF_10_ = 0.36; BF_01_ = 2.78, and no interactions containing test site (*F* ≤ .1.17, *p* ≥ .324; BF_10_ ≤ 0.10; BF_01_ ≥ 10.00).

The BF analysis indicated that the model with the highest likelihood included main effects of probe value and directed refreshing, as well as an interaction between them (BF_10_ > 10,000 relative to the null model containing participant only). This model had a BF of 2.77 in comparison to the next preferred model (which contained the same main effects and interaction plus a main effect of test site). The preferred model had a BF of 5.15 relative to a model excluding the interaction between probe value and directed refreshing.

To understand the interaction, three one-way repeated-measures ANOVAs were conducted to investigate whether the effects of directed refreshing differed depending on the probe value condition. A significant effect of directed refreshing emerged in the high-value condition, *F*(1.69, 66.01) = 13.19, *p* < .001, η_p_^2^ = .25; BF_10_ = 1,544.81, driven by significant differences between the cued and uncued conditions (*p* = .004; BF_10_ = 20.47) and the none-cued and uncued conditions (*p* < .001; BF_10_ = 291.00). There was no significant difference between the cued and none-cued conditions (*p* = .093; BF_10_ = 0.66; BF_01_ = 1.52). There was also a significant effect of directed refreshing in the equal-value condition, *F*(1.52, 59.28) = 19.27, *p* < .001, η_p_^2^ = .33; BF_10_ > 10,000, with significant differences between cued and uncued items (*p* < .001; BF_10_ = 1,000.73) and cued and none-cued items (*p* < .001; BF_10_ = 3,592.59). There was no significant difference between the none-cued and uncued conditions (*p* = .056; BF_10_ = 0.97; BF_01_ = 1.03). Finally, in the low value condition, there was a significant effect of directed refreshing, *F*(1.58, 61.51) = 28.95, *p* < .001, η_p_^2^ = .43; BF_10_ > 10,000, with significant differences between all three conditions (cued vs. uncued: *p* < .001, BF_10_ > 10,000; cued vs. none cued: *p* < .001, BF_10_ = 188.33; none cued vs. uncued: *p* < .001, BF_10_ = 445.05).

In the high-value condition, the BF for the difference between the cued and none-cued conditions provided only weak evidence of no effect (BF_10_ = 0.66, BF_01_ = 1.52). However, participants exhibited a lower mean recall error in the none-cued condition than in the cued condition when the item was worth a high value. As this goes against the direction predicted on the assumption of independent value and cueing effects, a one-tailed BF *t* test was conducted to investigate the strength of evidence that participants exhibited lower mean error in the cued condition relative to the none-cued condition under high value. There was strong evidence against this hypothesis (BF_10_ = 0.07, BF_01_ *=* 14.29).

The interaction was also broken down by examining whether an effect of probe value emerged in the directed refreshing conditions. There was a significant effect of probe value when the item tested was cued, *F*(1.52, 59.45) = 27.85, *p* < .001, η_p_^2^ = .42; BF_10_ > 10,000, with significant differences between the high-value and low-value conditions (*p* < .001; BF_10_ > 10,000), the high-value and equal-value conditions (*p* < .001; *BF*_*10*_ *=* 132.29), and the equal-value and low-value conditions (*p* < .001; BF_10_ = 597.01). There was also a significant effect of probe value in the none-cued condition, *F*(1.39, 54.36) = 68.42, *p* < .001, η_p_^2^ = .64; BF_10_ > 10,000, with significant differences between all three conditions (high vs. low: *p* < .001; BF_10_ > 10,000; high vs. equal: *p* < .001; BF_10_ > 10,000; equal vs. low: *p* = .004; BF_10_ = 8.36). Finally, there was a significant effect of probe value in the uncued condition, *F*(1.39, 54.17) = 44.41, *p* < .001, η_p_^2^ = .53; BF_10_ > 10,000, again driven by significant differences between all three of the probe value conditions (high vs. low: *p* < .001; BF_10_ > 10,000; high vs. equal: *p* < .001; BF_10_ > 10,000; equal vs. low: *p* < .001; BF_10_ = 229.12).

### Mixture modelling

**Fig. 3:**
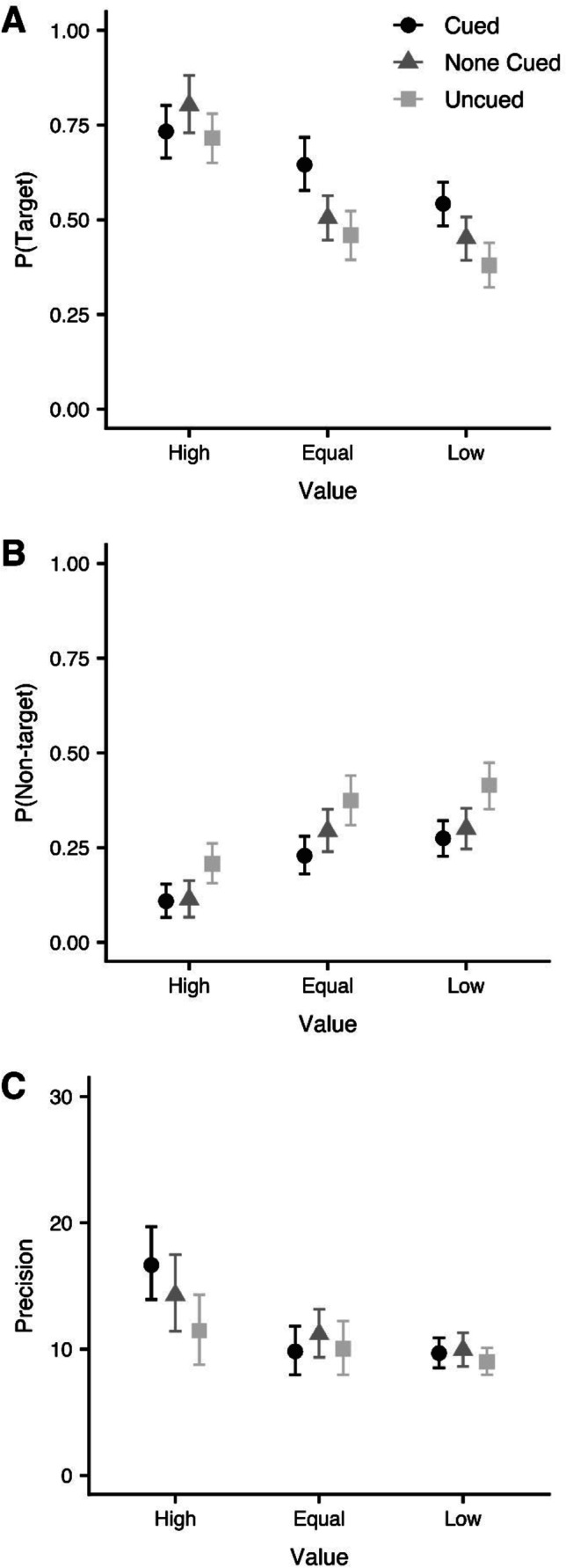
Estimates from the hierarchical Bayesian mixture modelling for the probability of recalling the target item (**a**), probability of recalling a nontarget item (**b**), and precision (**c**), as a function of probe value and directed refreshing. Points reflect the mean estimate, whereas the error bars reflect the 95% highest density intervals

As no differences were found across test site (i.e., either a main effect or any interactions), the data were combined for the mixture modelling. The parameter estimates from the hierarchical Bayesian mixture model are displayed in Fig. 3 as a function of probe value and directed refreshing.

**Fig. 4:**
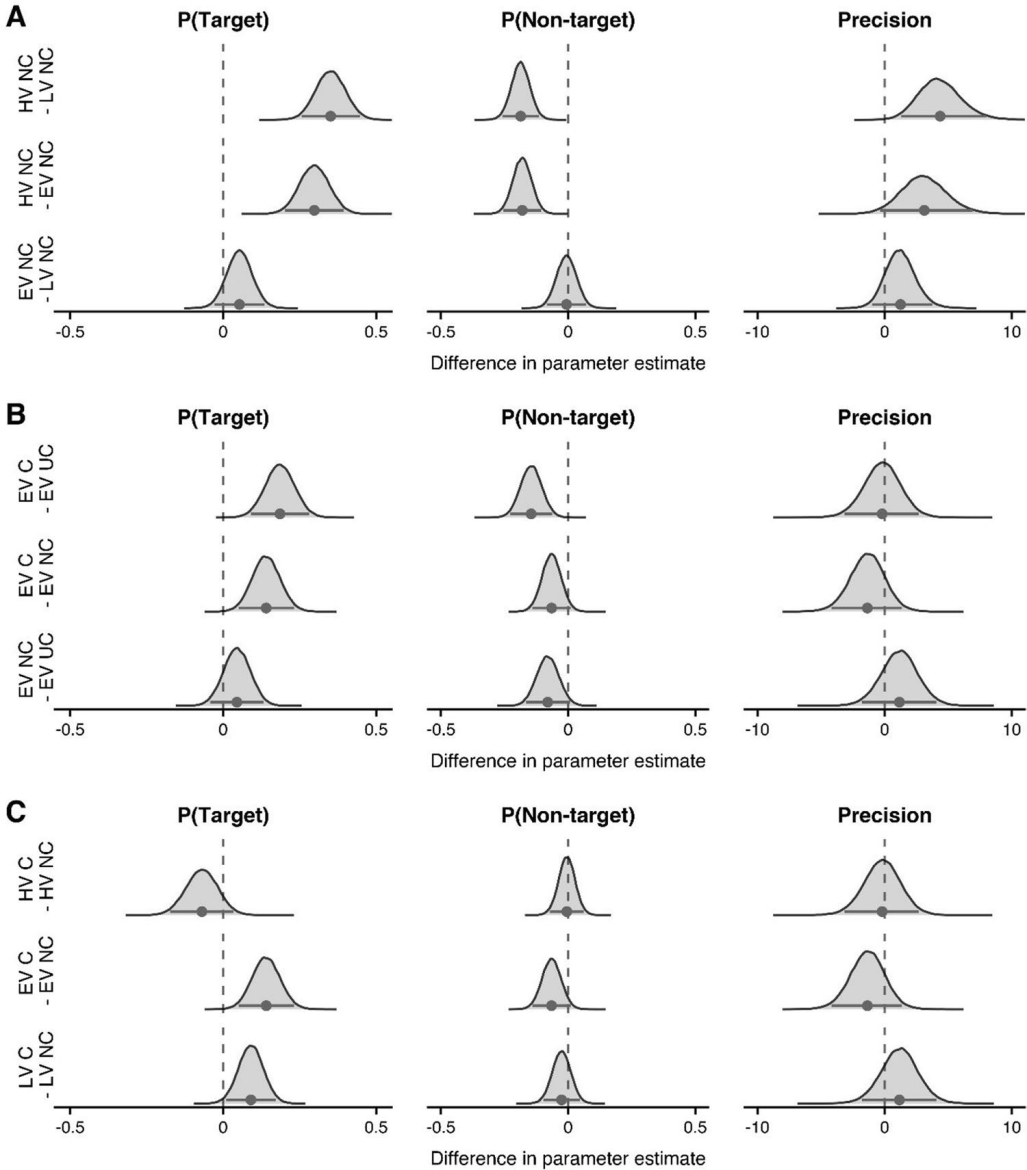
Differences between posterior distributions for the parameter in the hierarchical Bayesian mixture model (probability of recalling the target, probability of recalling a nontarget, and precision). Panel **a** presents the effect of probe value in the none-cued condition, panel **b** displays the effects of directed refreshing in the equal-value condition, and panel **c** presents the difference between the cue and none-cued conditions at each level of probe value. The differences were calculated by subtracting the posterior distribution of the second condition from the first. The first two letters of each facet reflect probe value (HV = high value; EV = equal value; LV = low value), whilst the second letters reflect the cueing condition (C = cued; NC = none cued; UC = uncued). The filled circles display the mean, the horizontal bars show the 95% highest density intervals, and the grey line at 0 reflects no difference

The difference in posterior distributions were calculated for the comparisons of interest. The mean, 95% highest density intervals, and distribution of these differences are displayed in Fig. 4. To explore the effect of probe value, the high-value, equal-value, and low-value conditions were compared in the none-cued condition (see Fig. 4a). The probability of recalling the target item was higher in the high-value condition than in the equal-value and low-value conditions. The probability of recalling a nontarget item was lower in the high-value condition relative to the low-value and equal-value conditions. Furthermore, responses were more precise for high-value items than for low-value items.

To explore the effect of directed refreshing, the posterior distributions for the cued, none-cued, and uncued conditions were compared on equal-value trials (see Fig. 4b). The probability of recalling the target item was higher in the cued condition than in the none-cued and uncued conditions. The probability of recalling a nontarget item was lower when items were cued relative to uncued.

As the primary research question was whether directed refreshing enhanced performance when the item was of high value, the posterior distributions for the cued and none-cued conditions were compared at each level of probe value (see Fig. 4c). The probability of recalling the target was higher in the cued condition than in the none-cued condition when the item was of equal or low value. Critically, no difference emerged when the item was of high value. There were no differences in the probability of recalling a nontarget item or in precision.

## Discussion

It has been proposed that the probe value effect in WM is driven by a biased attentional refreshing process (e.g., Atkinson et al., 2018; Atkinson et al., 2021; Hitch et al., 2020; Sandry et al., 2014), although no experimental studies have directly examined this. The current experiment tested this conjecture using the directed refreshing paradigm (Souza et al., 2015; Souza et al., 2018; Souza & Oberauer, 2017). We replicate the probe value and directed refreshing effects observed previously, with no notable differences between test sites. To the best of our knowledge, this study is the first to demonstrate the probe value effect using a continuous color reproduction task, with previous research using cued recall or recognition. Critically, an interaction between probe value and directed refreshing was observed. This was driven by lower recall error in the cued relative to the none-cued condition in the equal-value and low-value conditions, but no such effect in the high-value condition. Costs of cueing (i.e., poorer performance in the uncued vs. none-cued condition) were also observed in the high-value and low-value conditions.

Mixture modelling demonstrated that increasing the value of an item and directed refreshing both increasing the probability of recalling the target item. There was also some evidence that the manipulations decreased the probability of recalling a nontarget. However, one key distinction emerged: whilst probe value affected precision, cueing did not. Crucially, in line with the analyses examining recall error, cueing enhanced the probability of recalling the target item in the equal-value and low-value conditions, but not in the high-value condition.

In sum, encouraging individuals to refresh an item reduced recall error and enhanced accessibility in the equal-value and low-value conditions, but not in the high-value condition. This absence of a boost in the high-value condition indicates that individuals were likely to be already refreshing the more valuable item when no cues were presented. Further supporting the hypothesis that refreshing contributes to probe value effects, performance at the high-value item was reduced when refreshing was directed away from the high-value item. This shows the converse side of the coin: We cannot make people refresh high-value items more than they would spontaneously do, but we can prompt them to refresh them less, thereby reducing performance for these items. The current study therefore provides the first that attentional refreshing underlies the probe value effect in WM.

It might be argued that the absence of a cueing effect in the high-value condition would be expected if participants were unable to engage with both instructions simultaneously. However, if this was the case, one would expect no cueing effect to emerge when the item was associated with a low value, as individuals were still required to engage with both the probe value information and directed refreshing instructions in this condition. This was not observed, with cueing decreasing recall error and increasing accessibility in the low-value condition. As such, the current outcomes are more consistent with a biased attentional refreshing account.

Another possibility is that a cueing boost was not observed for the high-value item as performance in the high-value none-cued condition was near ceiling. However, this is unlikely, as mean recall error in this condition was considerably higher than the minimum (of 0°). Indeed, several studies using the continuous color reproduction task have demonstrated lower mean recall error than that observed in the current study (e.g., Arnicane & Souza, 2021; Oberauer & Lin, 2017; Souza et al., 2014). Further refuting this possibility, Atkinson et al. (2018) demonstrated that probe value and probe probability manipulations (whereby one item is more likely to be tested than the rest) are additive: Increasing the likelihood of an item being tested enhanced performance regardless of an item’s value. Whilst there were some differences between this task and the current study, this does indicate that memory for high-value items can be increased further. As such, the absence of additive effects in the current study cannot be attributed to an inability to further boost recall of high-value items.

Finally, it is possible that the high-value item may be somehow protected from forgetting, thus minimizing the benefits of directed refreshing. Whilst this could account for the lack of a cueing benefit in the high-value condition, this explanation would also predict the absence of cueing costs when other items are refreshed. However, as clear cueing costs emerged, we believe that the pattern of results observed is best explained by a biased attentional refreshing account.

We note that the process of refreshing induced by the cues could differ from the spontaneous refreshing that people apply to a high-value item. For instance, spontaneous refreshing could circulate faster from item to item (Vergauwe & Cowan, 2014), or it might consist of continuously focusing attention only on the high-value item (Oberauer & Lin, 2017). Such differences are immaterial to our argument as long as spontaneous and guided refreshing share two generally assumed characteristics of refreshing: Items compete for being refreshed at any point in time, and refreshing boosts an item’s availability in WM. As long as this is the case, our two predictions hold: Directing refreshing to a high-value item confers little additional benefit to it; directing refreshing to other items incurs a cost for high-value items.

These findings provide important insights into how the probe value manipulation might relate to other attentional manipulations, such as probe probability (e.g., Atkinson et al., 2018; Gorgoraptis et al., 2011). Evidence that the probe value effect is reliant on central attention (Hu et al., 2016), whereas the probe-frequency effect is relatively automatic (Atkinson et al., 2018), has been taken as evidence that these manipulations encourage individuals to direct attention in different ways. The current findings support this by demonstrating a potential mechanism by which probe value enhances WM. As probe probability effects are not dependent on central attention (Atkinson et al., 2018) whereas attentional refreshing is assumed to rely on this (e.g., Camos et al., 2018), it is unlikely that the probe frequency effect is driven by attentional refreshing.

Whilst our current findings support the biased attentional refreshing account, they do not suggest that the probe value effect is driven *entirely* by this process. As participants are told which item is more valuable prior to encoding, it is plausible that the effect partially reflects participants encoding the more valuable item differently than the other items (Allen & Atkinson, 2021; Sandry et al., 2014; Wang et al., 2017). This could explain why probe value enhanced the precision of the high-value items, whereas directed refreshing did not.

In summary, our results illuminate the mechanisms by which people can flexibly boost more valuable information in WM. First, high-value items seem to be encoded with higher precision than are low-value items. Second, high-value items are refreshed more during maintenance than are low-value items, thereby increasing their accessibility. Therefore, attentional mechanisms operating at both WM encoding and maintenance are likely needed to explain the probe value effect.

## Footnotes

1 Three participants from the University of Zurich did not engage with the “la” task to the appropriate standard, and recordings were not available for two participants. The outcomes reported here contains these participants’ data, but the main analysis was also conducted excluding these participants. There were no differences in the key outcomes after excluding these participants.

## References

[CR1] Allen RJ, Ueno T (2018). Multiple high-reward items can be prioritized in working memory but with greater vulnerability to interference. Attention, Perception & Psychophysics.

[CR2] Allen RJ, Atkinson AL, Nicholls LAB (2021). Strategic prioritisation enhances young and older adults’ visual feature binding in working memory. Quarterly Journal of Experimental Psychology.

[CR3] Allen, R. J., & Atkinson, A. L. (2021). Retrospective and prospective prioritization in visual working memory. PsyArXiv. 10.31234/osf.io/4x8zu

[CR4] Arnicane, A., & Souza, A. S. (2021). Assessing the robustness of feature-based selection in visual working memory. Journal of Experimental Psychology: Human Perception and Performance, 47(5), 731–758. 10.1037/xhp0000911 10.1037/xhp0000911 34264730

[CR5] Atkinson AL, Berry EDJ, Waterman AH, Baddeley AD, Hitch GJ, Allen RJ (2018). Are there multiple ways to direct attention in working memory?. Annals of the New York Academy of Sciences.

[CR6] Atkinson AL, Waterman AH, Allen RJ (2019). Can children prioritize more valuable information in working memory? An exploration into the effects of motivation and memory load. Developmental Psychology.

[CR7] Atkinson AL, Allen RJ, Baddeley AD, Hitch GJ, Waterman AH (2021). Can valuable information be prioritized in verbal working memory?. Journal of Experimental Psychology: Learning, Memory, and Cognition.

[CR8] Baddeley A (1986). *Working memory*.

[CR9] Bays PM, Catalao RF, Husain M (2009). The precision of visual working memory is set by allocation of a shared resource. Journal of vision.

[CR10] Camos V, Johnson M, Loaiza V, Portrat S, Souza A, Vergauwe E (2018). What is attentional refreshing in working memory?. Annals of the New York Academy of Sciences.

[CR11] Cowan N (2017). The many faces of working memory and short-term storage. Psychonomic Bulletin & Review.

[CR12] Gelman A, Rubin DB (1992). Inference from iterative simulation using multiple sequences. Statistical Science.

[CR13] Gorgoraptis N, Catalao RFG, Bays PM, Husain M (2011). Dynamic updating of working memory resources for visual objects. Journal of Neuroscience.

[CR14] Hitch GJ, Hu Y, Allen RJ, Baddeley AD (2018). Competition for the focus of attention in visual working memory: perceptual recency versus executive control. Annals of the New York Academy of Sciences.

[CR15] Hitch GJ, Allen RJ, Baddeley AD (2020). Attention and binding in visual working memory: Two forms of attention and two kinds of buffer storage. Attention, Perception, & Psychophysics.

[CR16] Hu Y, Hitch GJ, Baddeley AD, Zhang M, Allen RJ (2014). Executive and perceptual attention play different roles in visual working memory: evidence from suffix and strategy effects. Journal of Experimental Psychology: Human Perception and Performance.

[CR17] Hu Y, Allen RJ, Baddeley AD, Hitch GJ (2016). Executive control of stimulus-driven and goal-directed attention in visual working memory. Attention, Perception, & Psychophysics.

[CR18] Klyszejko Z, Rahmati M, Curtis CE (2014). Attentional priority determines working memory precision. Vision Research.

[CR19] Loaiza VM, Souza AS (2018). Is refreshing in working memory impaired in older age? Evidence from the retro-cue paradigm. Annals of the New York Academy of Sciences.

[CR20] Morey, R. D., Rouder, J. N., Jamil, T., Urbanek, S., Forner, K., & Ly, A. (2018). Package ‘BayesFactor’ (Version 0.9.12-4.2). Retrieved from https://cran.rproject.org/web/packages/BayesFactor/BayesFactor.pdf

[CR21] Oberauer K, Hein L (2012). Attention to Information in working memory. Current Directions in Psychological Science.

[CR22] Oberauer K, Lin H-Y (2017). An interference model of visual working memory. Psychological Review.

[CR23] Oberauer K, Stoneking C, Wabersich D, Lin HY (2017). Hierarchical Bayesian measurement models for continuous reproduction of visual features from working memory. Journal of Vision.

[CR24] R Core Team (2018). R: A language and environment for statistical computing. https://www.r-project.org/

[CR25] Rerko L, Souza AS, Oberauer K (2014). Retro-cue benefits in working memory without sustained focal attention. Memory & Cognition.

[CR26] Rouder JN, Morey RD, Speckman PL, Province JM (2012). Default Bayes factors for ANOVA designs. Journal of Mathematical Psychology.

[CR27] Sandry J, Schwark JD, MacDonald J (2014). Flexibility within working memory and the focus of attention for sequential verbal information does not depend on active maintenance. Memory & Cognition.

[CR28] Souza AS, Oberauer K (2016). In search of the focus of attention in working memory: 13 years of the retro-cue effect. Attention, Perception, & Psychophysics.

[CR29] Souza AS, Oberauer K (2017). The contributions of visual and central attention to visual working memory. Attention, Perception, & Psychophysics.

[CR30] Souza AS, Rerko L, Lin H-Y, Oberauer K (2014). Focused attention improves working memory: Implications for flexible-resource and discrete-capacity models. Attention, Perception, & Psychophysics.

[CR31] Souza AS, Rerko L, Oberauer K (2015). Refreshing memory traces: Thinking of an item improves retrieval from visual working memory. Annals of the New York Academy of Sciences.

[CR32] Souza AS, Vergauwe E, Oberauer K (2018). Where to attend next: guiding refreshing of visual, spatial, and verbal representations in working memory. Annals of the New York Academy of Sciences.

[CR33] Vergauwe, E., & Cowan, N. (2014). A common short-term memory retrieval rate may describe many cognitive procedures. *Frontiers in Human Neuroscience, 8*. 10.3389/fnhum.2014.00126 10.3389/fnhum.2014.00126 PMC3945934 24639643

[CR34] Vergauwe E, Langerock N (2017). Attentional refreshing of information in working memory: Increased immediate accessibility of just-refreshed representations. Journal of Memory and Language.

[CR35] Wang B, Yan C, Wang Z, Olivers CNL, Theeuwes J (2017). Adverse orienting effects on visual working memory encoding and maintenance. Psychonomic Bulletin & Review.

[CR36] Zhang, W., & Luck, S. J. (2008). Discrete fixed-resolution representations in visual working memory. Nature, 453(7192), 233-235. 10.1038/nature06860 PMC2588137 18385672

